# Diabetic Foot: The Role of Fasciae, a Narrative Review

**DOI:** 10.3390/biology10080759

**Published:** 2021-08-07

**Authors:** Carmelo Pirri, Caterina Fede, Nina Pirri, Lucia Petrelli, Chenglei Fan, Raffaele De Caro, Carla Stecco

**Affiliations:** 1 Department of Neurosciences, Institute of Human Anatomy, University of Padova, 35121 Padua, Italy; caterina.fede@unipd.it (C.F.); lucia.petrelli@unipd.it (L.P.); yutianfan1218@163.com (C.F.); rdecaro@unipd.it (R.D.C.); carla.stecco@unipd.it (C.S.); 2 School of Medicine and Surgery, University of Messina, 98125 Messina, Italy; nina_92_@hotmail.it

**Keywords:** wounds, fascia, diabetes, superficial fascia, stiffness, collagen, hyaluronan, diabetic foot

## Abstract

**Simple Summary:**

Diabetes mellitus and its complications are increasingly prevalent worldwide with severe impacts on patients and health care systems. Diabetic foot ulcers have an important impact on disability, morbidity, and mortality. The mechanism of diabetic wound chronicity has not yet been understood in a complete way. Regarding the involved soft tissues, little space has been given to the fasciae, even if nowadays there is more and more evidence of their role in proprioception, muscular force transmission, skin vascularization and tropism, and wound healing. Thus, we aimed to deepen the fascial involvement in diabetic wounds. Based on this review, we suggest that a clear scientific perception of fascial role can improve treatment strategies and create new perspectives of treatment.

**Abstract:**

Wound healing is an intricate, dynamic process, in which various elements such as hyperglycemia, neuropathy, blood supply, matrix turnover, wound contraction, and the microbiome all have a role in this “out of tune” diabetic complex symphony, particularly noticeable in the complications of diabetic foot. Recently it was demonstrated that the fasciae have a crucial role in proprioception, muscular force transmission, skin vascularization and tropism, and wound healing. Indeed, the fasciae are a dynamic multifaceted meshwork of connective tissue comprised of diverse cells settled down in the extracellular matrix and nervous fibers; each constituent plays a particular role in the fasciae adapting in various ways to the diverse stimuli. This review intends to deepen the discussion on the possible fascial role in diabetic wounds. In diabetes, the thickening of collagen, the fragmentation of elastic fibers, and the changes in glycosaminoglycans, in particular hyaluronan, leads to changes in the stiffness, gliding, and the distribution of force transmission in the fasciae, with cascading repercussions at the cellular and molecular levels, consequently feeding a vicious pathophysiological circle. A clear scientific perception of fascial role from microscopic and macroscopic points of view can facilitate the identification of appropriate treatment strategies for wounds in diabetes and create new perspectives of treatment.

## 1. Introduction

Diabetes mellitus (DM) and its complications are increasingly prevalent worldwide with severe impacts on patients and health care systems [1]. Diabetic foot ulcers have an important impact on disability, morbidity, and mortality. The mechanism of diabetic wound chronicity has not yet been understood in a complete way.

The Word Health Organization and International Diabetes Federation define chronic wound diabetes complications as diabetic foot, resulting in ulcers within the soft tissue due to a combination of neuropathy, peripheral vascular disease (ischaemia), and hyperglycaemia [2]. The pillar of treatment for these complications is addressing the extrinsic factors of repeated trauma, ischaemia and infection, and optimizing glycaemic control [3]. The intrinsic factors have been actively researched for the past three decades, including the molecular research of impaired healing in the diabetic wound, and in any case, this is not completely understood [3]. One of the key problems is that the term ‘soft tissues’ includes many different tissues, such as muscle, tendons, ligaments, fat, fibrous tissue, lymph and blood vessels, fasciae, and synovial membranes. Really, these tissues have different microscopic characteristics and respond in different ways to the various mechanical and metabolic inputs.

A fascia is morphologically “a sheath, a sheet, or any other dissectible aggregations of connective tissue that forms beneath the skin to attach, enclose, and separate muscles and other internal organs” [4]. In our body, many different types of fasciae can be recognized. Based on their histological features and anatomical relationships, the fasciae are divided into two types:

1. Superficial fascia: is located under the skin and superficial adipose layer, inside of the subcutaneous tissue [4]. It is a fibro-elastic layer that divides the subcutaneous tissue in two parts: the superficial adipose tissue (SAT) and the deep adipose tissue (DAT) [4].
2. Deep/muscular fasciae: are in contact with the muscles and are classified depending on their composition, orientation, and architecture as either aponeurotic and epimysial [4].

Fasciae are strongly influenced by the hormonal and endocannabinoid systems, and by mechanical and age factors [4], and there is some evidence that suggests that an altered microenvironment such as that of diabetes can also have a pathological impact on them [4]. Knowing all the factors that can act on the fascial structure, we can have a multidisciplinary targeted approach to patients with diabetic foot.

For this reason, the main purpose of this review is to understand the possible role of the fasciae in the pathology of the diabetic foot.

## 2. Role of the Deep Fasciae in the Diabetic Foot

**Figure 1:**
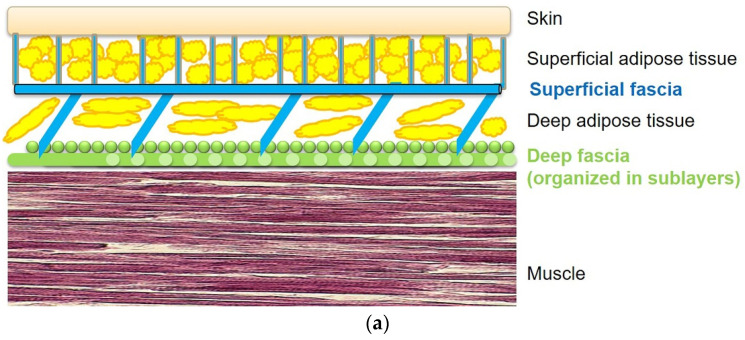

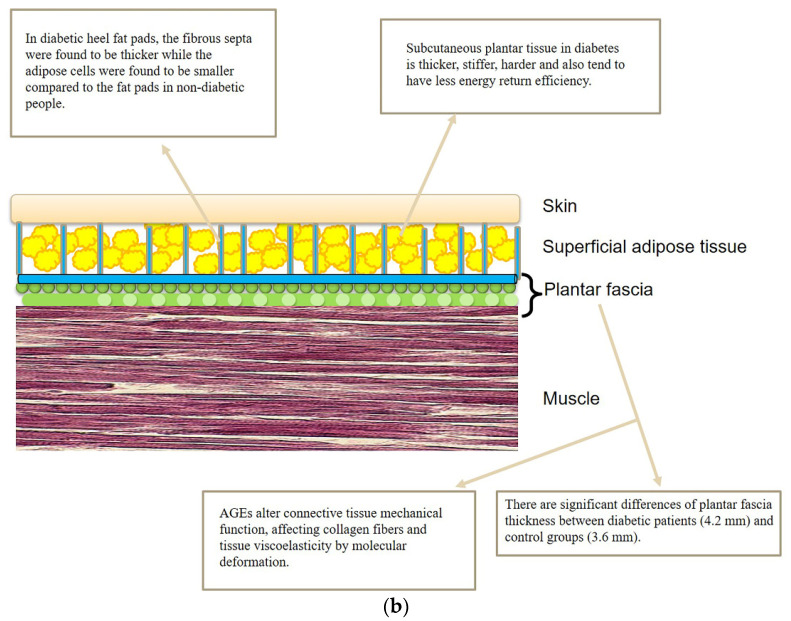
Schematic representation of the various fasciae from skin to muscle: (**a**) dorsum of the foot, (**b**) plantar region of the foot.

In the foot, we need to distinguish the fascial organization of the dorsum and of the plant. Indeed, in the dorsum we can recognized both a superficial and a deep fascia (Figure 1a), whilst in the plantar region, the superficial fascia and deep fascia are fused to form the plantar fascia (Figure 1b). Consequently, in the plantar region there is a physiological lack of gliding between the skin and the deep tissues, probably to permit the foot to adhere to the ground. Moreover, it is well known that the plantar fascia is well innerved [5] and it is capable of perceiving the foot’s position. Furthermore, it is well known that there are significant differences of plantar fascia thickness between diabetic and control groups [6] (Figure 1b).

Besides, the plantar fascia gives insertion to many muscular fibers of the intrinsic muscles of the foot, and is in continuity with the Achilles tendon. Consequently, if these muscles contract excessively, the plantar fascia, and the nerve endings it contains, might be overstretched, causing pain or a change in its mechanical behavior. Bolivar et al. identified a greater tightness of the posterior leg muscles in individuals with plantar fasciitis and suggested that therapists intending to use a stretching protocol to treat plantar fasciitis should look for both hamstring and triceps surae tightness [7].

Similarly, in diabetes, stiffness of the triceps surae muscle and foot muscles contracture concur with the start of ulceration. Ankle equinus is one of the most frequent causes of foot ulceration, as there is increased pressure on the foot plantar surface during different tasks, mostly during walking [8]. Among diabetes patients, it is thought that the occurrence of the triceps surae contributes to ulcer formation, increasing the risk of recurrent ulcers, nevertheless the traditional off-loading therapeutic strategies have failed [8]. Furthermore, initially the ulcer can be determined by external injuries but the prolonged static positioning, due to foot wounds, complicates the limb contracture and consequently the resulting equinus. The forefoot stress could be further increased by the presence of hammer toe and claw toe [9]. In the last stage, it is difficult to trace a precise causal determination: for example, if the contracture or the ulceration came first [10]. In this vicious circle the continuity between the calf and plantar regions through fasciae could be useful to better address the treatment. Indeed, fasciae could have an important role in the light of cellular and molecular alterations, explaining the macroscopic clinical complications, visible in daily clinical and rehabilitative practice (Figure 1b).

In cases with active diabetic foot ulcers, a statically positioned limb will cause contracture formation in the position of immobilization, due to fibrotic alterations within the muscles and fasciae. Therefore, the replacement of the muscle fibers with the collagen and fat of the deep fasciae, and the chronic shortening of the muscle length determine, in inactive diabetic patients, the onset of plantarflexion contracture. A very worrying situation happens when the soft tissue alterations begin before the onset of immobility. From a molecular point of view, the synthesis of protein in the muscle is decreased from 6 h after immobilization. In the first 24 h there is the shortening of the muscle fibers and 24 h later the perimysium begins to be infiltrated by collagen [10].

## 3. Role of the Superficial Fascia and Subcutaneous Tissue in the Diabetic Foot

In the same way, the subcutaneous plantar tissue in people with diabetes is thicker [11], stiffer [12,13], harder [14], and also has less efficient return of energy in case of stretching [15]. Indeed, some studies reported an increase of the fibrous septa thickness in the heel fat pads of diabetic patients, while the adipose cells were found to be smaller [16,17]. Really, according to Buschmann et al., in these septa the collagen and elastic fibers are fragmented and with an irregular deposition [16]. This evidence explains the harmful modifications of the superficial fascia’s mechanical properties. Furthermore, the alteration of collagen and elastin of the superficial fascia could lead to an alteration in the support and distribution of forces at the subcutaneous level with alteration of the microcirculation determined by an alteration of the activation triggers of short reflex arcs though the spinal cord and local reflex within the skin [18,19]. This causes a less elastic diabetic fat pad and, in turn, an impaired cushioning effect in distributing pressure [16] (Figure 1b).

## 4. Microscopic Organization of Foot Fasciae and Their Possible Role in Diabetic Foot

**Figure 2:**
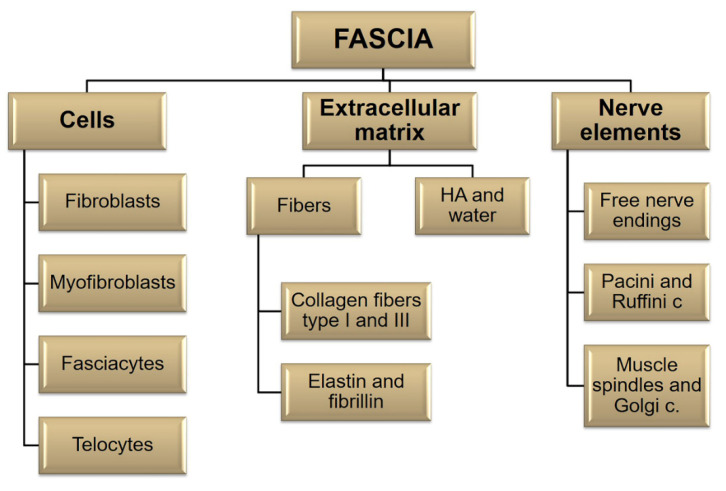
Schematic representation of the various fascial elements.

All the fasciae are composed of various kinds of cells embedded in an extracellular matrix and they are richly innerved. The nervous components define the sensitive role of the fascial tissue, whereas the cellular components adapt the fascia to varying conditions, defining the metabolic properties, and synthesize the extracellular matrix (Figure 2). These elements are present in different percentages in the various fasciae, defining the different mechanical and metabolic proprieties of each fascia. For example, the superficial fascia is more elastic and has more nerve elements [20] than the deep fasciae. The ankle retinacula have more hyaluronan with respect to the epimysial fasciae [21], and the plantar fascia seems to have a variable amount of HA, moreover, nobody knows the correct amount of HA in this fascia. All these elements could be involved in different ways in diabetic foot.

**Figure 3:**
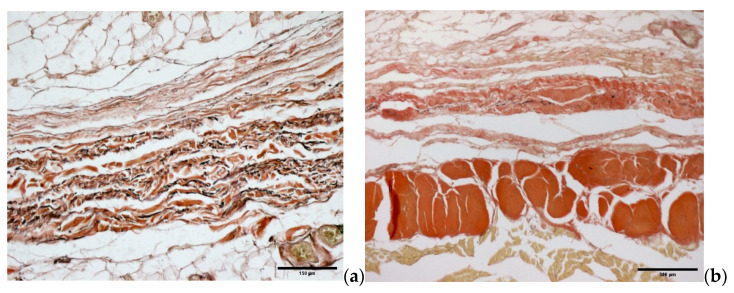
Histological images (Weigert Van Gieson staining that highlights the elastic and collagen fibers): (**a**) superficial fascia, (**b**) deep/muscular fascia.

The fascial cells are imbedded in a huge 3D extracellular matrix (ECM) consisting of an extremely dynamic matrix, made up of collagens, elastin, proteoglycans/glycosaminoglycans (GAGs), fibronectin, laminins, and other glycoproteins [4]. The types of molecules most present in the fascial ECM are fibrous proteins and a ground substance. The fibrous component is crucial for transmitting muscular force, connecting different segments and supporting vessels and nerves [22]. It is made up of collagen fibers (especially types I and III) and elastic fibers (elastin and fibrillin) [23] (Figure 3a,b).

**Figure 4:**
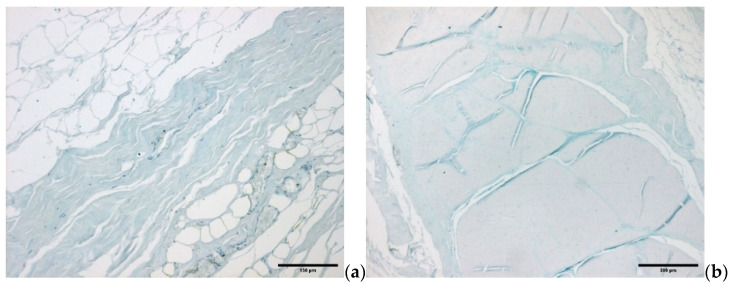
Alcian Blue/MgCl_2_ 0.05 M in sodium acetate buffer for glycosaminoglycans (GAGs) staining: (**a**) superficial fascia, (**b**) deep/muscular fascia.

The water component of the fascial ECM consists of a complex mixture of GAGs, almost always covalently linked to proteins, in the form of proteoglycans and glycoproteins. The most important GAG is hyaluronan [21] (Figure 4a,b).

### 4.1. Diabetic Foot: The Role of Cells

In diabetic wounds, many reparative processes are disrupted. Fibroblasts demonstrate a phenotypic modification as well as decreased migration and proliferation [24].

Fibroblasts are involved in many key processes such as dissolving fibrin clots, forming new ECM and collagen, and performing wound contraction. The exact origin of the fibroblasts is not clear, but they are usually recruited from the dermis, wound site or by phenotypic alteration of tubular epithelial cells via epithelial–mesenchymal transition (EMT) [25,26]. Recently, Correa-Gallegos et al. demonstrated that the fascial fibroblasts of the superficial fascia coordinate their action with the coagulation cascade, driving the production of a temporary matrix in wounds [27]. According to the authors, fasciae are a sort of deposit with an efficient mechanism to speedily seal large open wounds. Wan et al. [28], using live imaging of fascia fibroblasts and fate tracing of the fascia ECM, showed that “Connexin 43 (Cx43) expression is substantially upregulated in specialized fibroblasts of the fascia deep beneath the skin that are responsible for scar formation”, and the disruptions of these mechanisms has wide clinical implications in treating fibrosis, aggravated scarring, and impaired wound healing, such as in diabetic and ulcerative wounds. In the proliferative phase of the wound healing process, fibroblasts have an important role, migrating, proliferating, and producing ECM in response to various protein growth factors, e.g., epidermal growth factor (EGF), insulin, interleukin-1b (IL-1B), tumor necrosis factor-a (TNF-a), trasforming growth factor-β1 (TGF-β1), platelet-derived growth factor (PDGF), and fibroblasts growth factors (FGF) [29,30]. In tissue, fibroblasts attach to collagen fibrils in two different ways: (1) directly, by collagen receptors (e.g., a1b1 and a2b1 integrins) [31], and (2) indirectly via fibronectin by a5b1 integrin [32]. Moreover, the higher amount of collagen gels determines an important inhibitory effect on the fibroblasts’ proliferation, whereas when the collagen gel is denatured, it does not assemble into fibrils and does not inhibit the fibroblast growth [33,34]. Therefore, protein growth factors and the interaction with collagen fibrils check the fibroblasts growth. Jiang et al. [35] proposed a new model of scarring in which N-cadherin has a crucial role, as well as a sort of “cell swarming” that moves to scar centers. Fascia fibroblasts of the superficial fascia, defined as embryonic “engrailed past fibroblasts” (EPFs), upregulated N-cadherin upon wounding. The fascia mobilization into wounds and the skin contraction are triggered by the “swarming” of these cells, that consequently drives the scar formation. This phenomenon was absent in tissues such as oral mucosa in which the fascia is not present, and also when the N-cadherin was chemically or genetically inhibited. This new model of scarring indicates that the active contraction is determined from other elements outside of the wound and not by the temporary granulation tissue [36]. Indeed, a relaxation of the skin contraction is present in the situation of wound edge excisions, in which the “swarming” would be stopped [37]. Moreover, data, from in silico studies on kidney and lung fibrosis, demonstrated that, in these tissues, fibroblasts had a Cadherin-11 upregulation [38], and furthermore, that the fibroblast invasion during lung fibrosis is directly linked to the cadherin upregulation [39].

The fascial involvement into wounds has huge significance both in clinical and therapeutic settings. New strategies could be devised and developed, for example blocking the N-cadherin, to prevent or reduce scar formation [40].

Another type of cell contained in the fasciae are myofibroblasts which are specialized fibroblasts that regulate the fascial basal tone due to their contractile activity [41]. They are the activated repair cells that produce and organize the necessary ECM to re-establish tissue integrity. Abundant myofibroblasts can give hypertrophic scarring and contractures if they work for too long [41]. Other important cellular actors are the fasciacytes, a small cluster of rounded cells along the surface of each deep fascial layer specialized in hyaluronan (HA) synthesis [42], and the telocytes, called a “network in network” by Dawidowics et al. [43], due to their thin extensions (telopodes) that form a 3D communication system in the interstitial ECM. Both cell types may have a role in diabetes that will need to be studied in future research.

### 4.2. Diabetic Foot: Fascial ECM, Fibrous Component Role

Compromised ECM remodeling is a typical presentation of diabetic foot ulcers. In defective wound healing, the balance between ECM degradation, production and maturation tends to yield more degraded, non-soluble fibrils, resulting in a disorganized ECM network [44].

Different growth factors (PDGF, TGF-β, ecc.) are responsible for the collagen synthesis and decrease the degradation of ECM. This overexpression of profibrotic cytokines leads to fibroplasia, where the fibroblasts continue to produce connective tissue components leading to an excess of ECM [45]. The balance between ECM deposition and degradation therefore determines the amount of ECM within a wound; if the rate of synthesis of new collagen exceeds that which is degraded, the result is the fibrosis. Alterations in ECM are well-documented changes which occur during the fibrotic disease process in diabetes mellitus (DM). However, there is a controversial situation between the in vivo and in vitro conditions that underlines the role of the functional environment that can determine a decrease in the integrity of the skin [44].

The key fascial fibrous element is the collagen, having regulation of cell attachment and differentiation roles, and consequently providing elasticity and tensile strength to bones [4]. Types I and III are the most abundent collagens in fasciae and intramuscular connective tissue (IMCT), but other types are also present [46].

In general, the fibroblasts produce collagen in fasciae and IMCT, but in fibrosis conditions, other types of cells may also produce it, including myofibers, mesenchymal stem cells, inflammatory cells, or endothelial cells.

The excessive aggregation of ECM elements is present in fibrosis (e.g., in myopathies), including in fasciae, and occurs during aging [47], and diabetes, characterized by increased endomysium as well as perimysium [27]. Diet-induced insulin resistance (IR) leads to an increase in the expression of collagen I, III and IV [48]. Moreover, in a study by Berria et al. (2006), using a murine model and human patients, it was observed that the level of skeletal muscle (SM) collagen was higher when insulin resistance (IR) was present [49]. In a comparative study, total collagen content was observed to increase in obese insulin-resistant individuals compared with lean individuals [50].

SM undergoes diabetes-induced modifications in the structure of the basement membrane (BM) and in the activity of the collagen synthesizing enzymes. A microarray analysis of the SM transcriptome in streptozotocin-induced diabetic mice showed a decrease in the gene expression of various collagen types (I, III, IV, V, VI, and XV). Furthermore, in diabetic muscles, an increase of the mRNA expression of various non-collagenous proteoglycans and glycoproteins was reported [51].

Arkkila et al. [50] demonstrated that in diabetic subjects the synthesis of the collagen type III fibers increases, whereas the synthesis of collagen type I fibers decreases.

Moreover, it remains an open question about whether the ECM’s role in the stiffness of these tissues is due to alterations in the ECM’s molecular composition, in the molecular features of collagen (e.g., formation of advanced glycation end-products (AGEs), or the accumulation of larger collagen quantity resulting in ECM thickening).

In diabetes patients, the ECM turn-over is affected by chronic hyperglycemia, determining different complications due to both metabolic and hemodynamic pathways [52]. Hyperglycemia determines the formation of high levels of AGEs, which directly boost the production of ECM components [53]. AGEs include a huge group of post-translational protein adducts and cross-links that can change proteins’ physical properties and adversely affect their function. Several studies have related AGEs to compromised connective tissue mechanical function, and focused on AGE-related alterations in collagen fiber mechanics [51]. AGEs can dramatically affect collagen tissue viscoelasticity and molecular deformation [51]. Fessel et al. [54] reported that AGEs determine mechanical effects at different hierarchical levels of tendons in the collagen (i.e., from the tissue to fiber, to fiber–fiber interactions, to fibril–fibril interactions, and ultimately at the sub-fibril scale) [55]. At the tissue level in ribose cross-linked specimens an evident loss of stress relaxation behavior was found [55]. This decrease in viscoelasticity can be ascribed to modifications at microscopical levels (i.e., the fiber and fibril levels). The glycation originally affects fibril–fibril and fiber–fiber sliding and the surface of the fibrils. The modification of fibril surface features and behavior affect the cell–collagen binding and the associated focal adhesion mediated signalling, the stretch activated ion channels (SACs) behavior, and sliding activation of the primary cilium, as well as the way the nucleus deforms under mechanical stress [56]. These findings might be the same in the fasciae, as the collagen organization is the same as in the tendons. Therefore, it is possible that changes in the ECM may have adverse effects on cell-mediated tissue homeostasis and the repair of fascia and overlying skin.

Additionally, AGEs interact with the renin angiotensin aldosterone system (RAAS) in a significant way. Angiotensin II, the principal physiological effector molecule of the RAAS, promotes fibrosis by stimulating ECM synthesis. In general, RAAS is known to contribute to the pathogenesis of diabetic micro- and macrovascular complications by causing varied tissue responses, involving not only fibrosis but also vasocontraction, inflammation, oxidative stress, and cell hypertrophy and proliferation [49]. Pathogenesis of fibrosis is also influenced by TGF-β that regulates the expression of various ECM proteins, acting as the principal pro-fibrotic element in diabetic nephropathy [56].

Li et al. (2013) highlighted that the major mechanical effect of AGEs is “a loss of tissue viscoelasticity driven by matrix-level loss of fiber-fiber sliding. This has potentially important implications for the accumulation of tissue damage, mechanically regulated cell signalling, matrix remodelling” [57].

Gautieri et al. (2017) demonstrated that the glycation of the collagen fibers decreases tissue viscoelasticity by severely limiting fiber–fiber sliding [58].

### 4.3. Diabetic Foot: Fascial ECM, Water Component Role

After injury, in wound repair tissue, the hyaluronan (HA) is elevated in concentrations for some weeks [59]. It is primarily produced by keratinocytes, but other cells such as fibroblasts can also produce it. In wound repair, among other functions, the HA provides a scaffold for cell migration to the damage sites, e.g., hepatic stellate cells that utilize CD44v6 to migrate toward a liver epithelial injury [60]. The expression of CD44 increases, as in the case of healed wounds, it seems to mimic the HA deposition pattern. It is as if the interaction between HA and CD44 provides a scaffolding on which tissue repairs itself. Other studies showed that low molecular mass and high molecular mass HA found in sites of injury may have direct effects on regeneration. Tolg et al. [61] demonstrated how important the HA size is to the final effect, i.e., they highlighted that six HA oligomer fragments promote different actions (e.g., wound closure, M2 macrophages accumulation, TCF-β1 production with accumulation of M2 macrophages, and production of TGF-β1 without causing myofibroblast differentiation). On the contrary, 40 kDa HA fragments have the opposite effect (e.g., wound closure inhibition and myofibroblasts accumulation/differentiation). Some years ago, a new cell devoted to HA production was described in the fasciae, including in the plantar fascia, and for that reason it was called a fasciacyte. It is a rounded cell specialized in hyaluronan (HA) synthesis, placed along the surface of each deep fascial layer [42]. The production of hyaluronan in the fascial ECM can be affected by many physical, mechanical and metabolic factors, and these can affect the hydration level of the fasciae [42]. “The amount of HA varies according to the anatomical site and to the fascial type: in the aponeurotic fasciae it is about 43 μg/g, but it drastically decreases (about 6 μg/g) in epimysial fasciae, and it increases in the retinacula (90.4 μg/g)“ [62]. These variations correlate with the different gliding fascial functions changing in the various anatomical sites: the aponeurotic fasciae, i.e., the rectus sheath of the abdomen and the fascia lata of the thigh glide freely over the muscles, whereas the epimysial fascia is a fibrous layer adherent to the underlying muscles with lower capacity for gliding [63]. Specialized aponeurotic fasciae are the retinacula which surround the joints, and they have the highest levels of HA [64]. The amount of HA in fasciae could be modulated by the endocannabinoid system. Lately, Fede et al. [65] proved that human fascial fibroblasts, in vitro, produce HA-rich vesicles after a few hours of CB2 receptor agonist treatment, resulting in greater tissue fluidity.

HA has a crucial role, not only in regulating the fascial features, but also in cell proliferation and mobility, inflammation, and angiogenesis. Moreover, various papers highlighted the crucial role in diseases such as cancer and diabetes [21,66]. The variability in its biological functions depends on the molecular weight of the HA. Indeed, HA polymers have dimensions from few kilodaltons to 8 MDa [67]. In general, depending on the molecular weight, HA polymers can have immunosuppressive, anti-inflammatory, antiangiogenic, and tissue damage repair properties at high weights, and on the contrary, proinflammatory and proangiogenic actions for the smaller fragments. The Ha viscosity can be altered due to changes in chemical and physical parameters (i.e., changes in the temperature and the hydration). A decrease in viscosity can be caused by an increase of two degrees centigrade in temperature (e.g., due to massage) with consequential gradual break-up of the 3D HA chains superstructure [68]. After intense physical exercise, the opposite can happen, because the muscle can reach a pH value of about 6.60, with a consequent increase in viscosity (approximately 20%) [69].

The interaction between HA and CD44, and the HA size and longevity are crucial for the process of wound healing. Future research into HA size and location may help medical therapies and surgical interventions, possibly by the use of an HA medical device to avoiding surgical scarring by introducing small HA oligomers (with short biological half-life) in the fascial layers after the wound incision, preventing the fibrosis and inflammation [70].

### 4.4. Diabetic Foot: Fascial Nerve Elements Role

Neuropathy leads to a loss of sensitivity with consequent decreased perceptions of foot trauma and development of foot ulcers. Traditionally, diabetic neuropathy (DN) is classified into diffuse and focal types, affecting the somatic or the autonomic part of the peripheral nervous system. In the latter case, it is classified as diabetic autonomic neuropathy (DAN) with greater involvement at the cardiovascular and gastrointestinal level. However, the most common form of DN is diabetic peripheral neuropathy (DPN) [71,72]. The clinical presentation of DPN is typically characterized by a progressive symmetrical sensory loss (e.g., temperature, pain, light touch and vibration perception) in the extremities of the lower limbs (so called “stocking and glove distribution”) [71,72]. Later, the diabetic patients experience motor deficits (e.g., muscle weakness, attenuation of reflexes). Furthermore, the skin can become fry and fragile due to the involvement of sweat gland innervation. At the beginning most patients have no symptoms, however the combination of these symptoms puts the feet at greater ulceration risk due to unrecognized trauma [71,72].

Until now, the free nerve endings and the autonomic fibers involved in diabetic neuropathy are seen as isolated structures, but in reality, they are totally embedded in the superficial and deep fasciae. Numerous authors have demonstrated the fascial large thin network of free nerve endings which have a crucial role in pain perception and regulation. The superficial fascia is the second most highly innervated tissue in the hip, after the skin [20]. The fasciae are also connected to autonomic innervation. Mense highlighted that approximately 40% of the entire fascia innervation consist of postganglionic sympathetic fibers [73]. The diabetic fascial dysfunction could occur before direct nerve involvement, which will need to be investigated in the future. Kumar et al. (2015) observed a significant reduction of the plantar fascia thickness in diabetes mellitus type 2 subjects with and without PN compared with non-diabetes mellitus subjects. The plantar fascia changes appear before the develop of peripheral neuropathy [74].

Diabetes duration, marked hyperglicemia, dyslipidaemia, hypertension, smoking, and age are the principal risk factors of DN [71]. The DN pathogenesis is an incompletely understood and complicated puzzle, in which every variable has its weight, e.g., hyperglycaemia, insulin resistance, chronic inflammation, oxidative stress, dyslipidaemia, endothelial dysfunction, and others [71,72]. Currently, the accumulation of AGEs in peripheral nerves is thought to be an additional cause, by increasing reactive oxygen species (ROS) that determine neural inflammation and consequently impair axonal transport. Moreover, the accumulation of AGEs has been correlated with a decrease or loss of sensitivity in lower limbs [75]. They also alter the surrounding connective tissue, particularly by affecting collagen fibers with molecular deformation and changing viscosity [51].

Identifying the precise cause is hard and complex; however, this is critical for diabetic foot management and the prevention of complications.

The innervation of the different structures involved in diabetic wound healing is crucial in the symptomatology and in the management of the patients. Indeed, skin innervation, by numerous neuromediators, is involved in all phases of wound healing [76]. It has hypothesized that there are a greater number of nerve fibers in excessive scarring compared to mature, linear scars [76]. Moreover, Substance P is the main neuromediator, with a central role in the process of inflammation releasing inflammatory cytokines, in upregulating fibroblasts, keratinocytes, and endothelial cell proliferation, and in remodelling, influencing collagen degradation by modulation of metalloproteinases (MMPs) on fibroblasts. Within each phase of wound healing, there are also other neuromediators: calcitonin gene-related peptide (CGRP); nerve growth factor (NGF); gastrin-related peptide (GRP); galanin; etc. that have different molecular signaling interactions.

The fasciae, like other tissues involved, are richly innervated [77] by peptidergic and non-peptidergic nociceptors [78]. Taguchi et al. showed in that deep fascia there are both Aδ and C types of free nerve endings [79]. Fede et al. found that the innervation of the various layers in the topographical region of the hip is different, demonstrating that the superficial fascia is the second most highly innervated tissue in the hip, after the skin [20]. All that suggests that the two types of fasciae have different amounts of innervation and consequently different roles. The deep fascia has a proprioceptive role, whereas the superficial fascia has a function in the exteroception. Sensorimotor diabetic polyneuropathy (DPN) affects both large and small sensory afferent nerve fibers [80]. The sensorimotor deficits resulting from large-fiber DPN, while sometimes subtle in nature, can lead to significant impairment. Muscle spindles, found within skeletal muscle, are embedded in the intramuscular connective tissue that is in continuity with the deep fasciae that already have their own innervation and, moreover, by their tensional state, modulate the muscle spindles. Indeed, Muller et al. identified reversible behavioral and pathological large sensory nerve fiber-related changes in diabetic mice [80]. Alteration in the proprioception of the diabetic patients due to fascial dysfunction could occur before direct nerve involvement, which will need to be investigated in the future. However, alteration of the superficial fascia innervation could also occur earlier with ambiguous symptomatology. Moreover, a subcutaneous tissue that is in an inflamed, glycated, stiffened, and fibrotic state will not provide the correct growth factors to the skin, leading to chronic inflammation without the regeneration and correct remodeling of the skin. A pathological fascial innervation in diabetes could explain a symptomatology with functional sensorimotor deficit.

## 5. Conclusions

In diabetic research there has been notable progress, but to date, diabetes complications management is restricted to treatment of the symptoms. Incomplete understanding of the entire molecular and cellular mechanisms underlying diabetes determines the low success of therapeutic strategies. Identification of potential risk factors, early diagnosis, and defining effective therapeutic strategies are some ongoing challenges for clinicians and researchers. New imaging techniques will be crucial for disease detection, while it will be very important to investigate how the modification of the risk factors of the disease can decrease risk of developing of diabetic complications, mostly in the area of prevention and prophylaxis. In this direction, this review highlights the necessity to study in a more systematic way the fascial structures in diabetic foot, focusing on the cell signaling and ECM mechanical behavior. The superficial and deep/muscular fasciae cells have different properties responding to the same signals; thus, the next challenge will be to precisely identify the cells’ roles and how to properly address them. Besides, it could be important to quantify the HA in the fasciae of diabetic patients, by evaluating a larger number of samples. Fascial changes, unlearned during clinical assessment, could be discovered thanks to a better molecular and cellular knowledge. A comprehensive clinical examination including the neurological, vascular, dermatological, and musculoskeletal systems should include an assessment of the fasciae that might be crucial for the early identification of changes occurring mostly in the high-risk foot, to prevent and manage the changes. Finally, defining the precise molecular and cellular elements affected in diabetic fasciae would help to create a more targeted approach to translate into treatments and therapies.

Diabetes researchers should adopt with greater urgency the recent major advances in technologies, integrating big data analysis and computational modelling. We are sure that, by integrated research efforts and by combining multidisciplinary knowledge, expertise, and skills, we will soon identify the components targetable by drugs/treatment that neutralize diabetic complications or even boost diabetic wound healing. Furthermore, breakthroughs in cellular therapy and creation of 3D fascial simulations for implementation in regenerative medicine, shall begin a new era of optimized clinical outcomes in diabetic complications management, finally adding an important missing piece to the complete knowledge of diabetes.

## Data Availability

Not applicable.
